# Survey on security issues of routing and anomaly detection for space information networks

**DOI:** 10.1038/s41598-021-01638-z

**Published:** 2021-11-15

**Authors:** Ming Zhuo, Leyuan Liu, Shijie Zhou, Zhiwen Tian

**Affiliations:** School of information and software engineering, University of Electronic Science and Technology of China, Chengdu, 610054 People’s Republic of China

**Keywords:** Information technology, Computer science, Aerospace engineering

## Abstract

Space information networks is network systems that can receive, transmit, and process spatial information lively. It uses satellites, stratosphere airships, Unmanned Aerial Vehicles, and other platforms as the carrier. It supports high-dynamic, real-time broadband transmission of earth observations and ultra-long-distance, long-delay reliable transmission of deep space exploration. The deeper the network integration, the higher the system’s security concerns and the more likely SINs will be controlled and destroyed in terms of cybersecurity. How to integrate new IT technologies such as artificial intelligence, digital twins, and blockchain to diverse application scenarios of SINs while maintaining SIN cybersecurity will be a long-term critical technical issue. This study is a review of the security issues for space information networks. First, this paper examines space information networks’ security issues and figures out the relationship between the main security threats, services, and mechanisms. Then, this article selects secure routing and anomaly detection from many security technologies to conduct a detailed overview from two perspectives of traditional methods and artificial intelligence. Subsequently, this paper investigates anomaly detection schemes for spatial information networks and proposes a deep learning-based anomaly detection scheme. Finally, we suggest the potential research directions and opening problems of space information network security. Overall, this paper aims to give readers an overview of the newly emerging technologies in space information networks’ security issues and provide inspiration for future exploration.

## Introduction

The Space Information Networks (SINs) is new network infrastructure, which uses satellites, stratospheric airships, Unmanned Aerial Vehicles (UAV), and other platforms as carriers to acquire, transmit and process spatial information in real-time. It is a hybrid network system that interconnects spatial satellite networks, modern communication networks, terrestrial Ad-Hoc, and the Internet. The principle of maximizing the effective use of spatial information resources provides reliable information services and comprehensive guarantees for customers with different spatial locations and business needs^1^. SINs support highly dynamic, the real-time broadband transmission of earth observation and ultra long-distance, long-delay reliable message transmission of deep space exploration. The main application scenarios of SINs include disaster emergency communications, data transmission in remote areas, marine operations and scientific research broadband, aviation broadband, special military missions, etc^2^.

SINs is different from the traditional terrestrial networks or tracking and data relay satellite system, and it is a heterogeneous integrated information network. Therefore, it has several unique characteristic features: heterogeneous network integration, dynamically changing network topology, extraordinarily long and variable propagation latency, asymmetrical forward and reverse link capacities, high bit error rate, intermittent link connectivity, lack of fixed communication resources, good network scalability, etc. These characteristics are very similar to Delay Tolerant Network (DTN), so the SINs can be seen as a typical DTN. However, because these characteristics are not common in the traditional ground network and make the network system more open, they bring new challenges for securing SINs. The first detected attack against SINs security was around 2007^3^, but the number of reported attacks has increased in recent years continuously. Some of the notorious incidents are listed as follows.

- In 2013, Somebody hacked an emergency alert system of TV stations in Montana and Michigan, and the attackers broadcast a fake report of a Zombie invasion^4^.
- In September of 2015, a cyber-espionage organization that exploits the Turla malware used satellites to achieve greater anonymity, according to new research from Kaspersky Lab^5^. The organization exploited security weaknesses in global satellite networks as part of its tradecraft. Hackers hid exfiltrated material in legit data streams of innocent users.
- In June of 2018, a sophisticated hacking campaign launched from computers burrowed deeply into satellite operators, defense contractors, and telecommunications companies in the United States and southeast Asia, as stated by security researchers at Symantec Corp^6^. The hacker could use infected computers that controlled the satellites to have changed the orbiting devices’ positions and disrupted data traffic.
- In 2019, U.S. Government Accountability Office reported to Congress that the U.S. navy’s next-generation narrowband tactical communication system, the Mobile User Objective System (MUOS), has pending security vulnerabilities. Military satellite systems also face security threats.
- In August 2020, the Department of the U.S. Air Force and the Defense Digital Service’s (DDS’s) hosted the Space Security Challenge 2020: Hack-A-Sat at the prestigious DEF CON hacking conference, allowing hackers to conduct hacking competition events against real satellites. By the end of the competition, six of the eight participating teams had completed the invasion challenge.

Existing research focuses on secure handover, secure transmission control, lightweight cryptographic algorithms, cross-domain key distribution and management, efficient access authentication, etc. Those work has been complete and sufficient. That is, little work^7,8^ about security routing and anomaly detection in space networks has been discussed.

This paper focuses on the security issues and countermeasures in SINs. We are attempting to link specific security concerns(such as security routing and anomaly detection) with machine learning technology, and we are devoting significant research resources to this new field. After reviewing the current research results, this paper also puts forward a design scheme aiming at the security challenges and existing problems. To summarize, this paper presents a comprehensive survey of newly emerging security technologies with the following contribution.

- Security threats in SINs are classified and enumerated. We collate the attacks and sabotages that have been reported so far. We introduce the security service and security mechanism of SINs and sort out the relationship between security threat, security service, and security mechanism.
- Secure routing technologies are summarized. We first analyze some significant problems that SINs routing faced. Then we classify routing from different angles, mainly from the perspective of the routing domain and network layer. Finally, we introduce intelligent routing technologies combination of machine learning that has been popular in recent years.
- Anomaly detection of dynamic network and SINs is reviewed. We give a broad overview of the related work in anomaly detection approaches used in a dynamic network because SINs is a particular dynamic network. We continue with an extensive overview of the existing anomaly detection methods in SINs. After that, we also proposed an anomaly detection scheme for SINs based on graph embedding.
- For enlightening future research, the potential future directions and open questions are sorted out. There are holistic security frameworks, integrated space-air-ground computing architecture, detailed security technologies, and virtual simulation platforms.

## Threats, security services and mechanisms in SINs

### Threats in SINs

**Figure 1:**
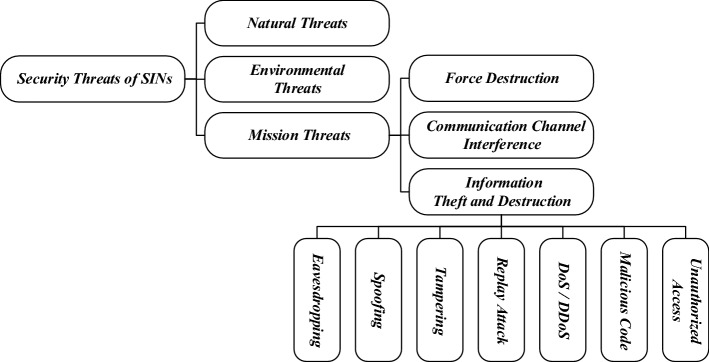
The SINs contain three types of threats: natural threats, environmental threats, and mission threats.

Compared with the traditional terrestrial network, SINs is more open and vulnerable to various attacks. Therefore, the privacy and security issues of SINs have attracted extensive attention. The Consultative Committee for Space Data Systems (CCSDS) report titled ”Security Threats against Space Missions”^9^ presents an overview of threats against space missions, including illustrative examples of threats against various classes of missions. As shown in Fig.1, the threats of SINs can be divided generally into three types: natural threats, environmental threats, and mission threats. Natural threats refer to threats caused by irresistible natural factors, such as interference from space electromagnetic radiation and severe natural climate. Environmental threats are understood to mean threats caused by the system operating environment failures, such as power outages, downtime. Threats created by organizations or individuals for various goals and motivations, such as malicious cyber-attacks, are referred to as mission threats. It includes force destruction, destruction or interference for communication channels, information theft and destruction for entire networks.

From an aerospace cyberspace security perspective, more than 50% of the attacks on SINs fall under the information theft and destruction. However, this paper only analyzes security threats in the hybrid SINs from information theft and destruction, for lack of space, will not discuss other types of threats. Common threats from information theft and destruction include eavesdropping, spoofing, tampering, replay attack, denial of service/distributed denial of service, malicious code and unauthorized access. Those security attacks, which similar to cyber-attacks on the Internet, can be launched against different hybrid satellite network topologies. He et al.^10^ establishes the attack model and attack simulation for a satellite platform based on the MIL-STD-1553B bus as an example. In addition, SINs is vulnerable to side-channel attacks due to using the same key for a longer duration.

### Security services and mechanisms in SINs

The information interaction among nodes for SINs is vulnerable to attacks such as eavesdropping, spoofing, tampering, replay, and DDoS/DoS attacks. In order to avoid these security threats, the network should meet basic security and efficiency requirements, that is, data integrity, confidentiality, truth, availability, and non-repudiation. Table 1 lists the relationship between these threats and requirements.

**Table 1:** The relationship between security threats and security requirements in SINs .

Security threats	Confidentiality	Integrity	Truth	Availability	Non-repudiation
Eavesdropping	$\surd$				
Spoofing			$\surd$	$\surd$	
Tampering		$\surd$		$\surd$	
Replay attack		$\surd$			$\surd$
DoS/DDoS					$\surd$
Malicious code		$\surd$		$\surd$	
Unauthorized access			$\surd$		$\surd$

Eavesdropping attack breaks confidentiality. Spoofing attack endangers truth and availability. Tampering attack curtails data integrity and availability. Replay attacks break non-repudiation. Malicious code breaks data integrity and availability. Unauthorized access jeopardizes the truth and non-repudiation of information.

The network security architecture designed by security threats and security requirements can regulate and guide network security services and implement security mechanisms. International Organization for Standardization standard IOS 7498-2 describes the architecture of open system interconnection security^11^. It proposes five security services and eight security mechanisms that can support security services. Network security architecture should provide five primary security services: authentication, access control, data confidentiality, data integrity, and non-repudiation. Table 2 shows the main security threats of SINs and the related security services and security mechanisms. The table’s columns represent the eight security methods. Security threats and security services are the two dimensions of its rows. Table 2 demonstrates the relationships between the seven security threats, five security services, and eight security measures. It can be seen from Table 2 that security mechanisms such as encipherment, digital signature, access control, data integrity, routing control can ensure that SINs do not suffer from most security threats. However, these security mechanisms rely on traditional security technologies such as cryptography, privacy protection, routing algorithms, and anomaly detection. In recent years, with the rise of artificial intelligence, especially, researchers turn their attention to intelligent security technology. Because of space constraints, we can not set out all of the mechanisms in the article. The following sections of this paper will investigate emphatically routing and anomaly detection technology as an example in SINs.

**Table 2:** The main security threats of SINs and the related security services and security mechanisms .

Security mechanisms		Encipher -ment	Digital signature	Access control	Data integrity	Authentication exchange	Traffic padding	Routing control	Notarization
Security threats	Security services								
Eavesdropping	Data confidentiality	$\surd$					$\surd$	$\surd$	
Spoofing	Authentication	$\surd$	$\surd$			$\surd$			
	Access control			$\surd$				$\surd$	
Tampering	Data Integrity	$\surd$	$\surd$		$\surd$				
	Access control			$\surd$				$\surd$	
Replay attack	Data Integrity	$\surd$	$\surd$		$\surd$				
	Non-repudiation		$\surd$		$\surd$				$\surd$
DoS/DDoS	Access control			$\surd$				$\surd$	
Malicious code	Access Control			$\surd$				$\surd$	
	Data integrity	$\surd$	$\surd$		$\surd$				
Unauthorized access	Authentication	$\surd$	$\surd$			$\surd$			

## Secure and intelligent routing issues in SINs

SINs confront a slew of security issues, the most notable of which may be routing security. SINs are likely to be paralyzed if routing is targeted by the malicious activity stated in the context. Therefore routing is essential to SIN security. Three security goals must be met while routing on SINs. The first goal is to examine the routing message authentication. The purpose of message authentication is to guarantee that the information is not tampered with during network transmission, assuring the routing information’s legitimacy and dependability. Because the SINs channel is open and attackers might intercept vital data during transmission, the second security aim of SINs routing is confidentiality to prevent data from leaking to non-transmitting nodes and successfully resist external eavesdropping attempts. The integrity of routing messages is the third security aim to examine. To steal transmission messages, attackers often employ methods such as removing transmission nodes or deliberately altering transmission routes, resulting in incomplete information. The integrity of the message propagation path and messages must be ensured by a secure SINs routing mechanism.

Many factors such as continuous dynamic change of topology structure, extension of data round trip, uneven distribution of data traffic, and complicated space environment, ground routing algorithm are not suitable for SINs anymore^12^. Therefore, this section focuses on the secure and efficient routing mechanism for the characteristics of the SINs. It reviews the related works of combining the traditional routing mechanism with the artificial intelligence method to ensure the integrity and trustworthiness of space information, as well as the availability and dependability of space services.

### Types of SINs’ routing

**Figure 2:**
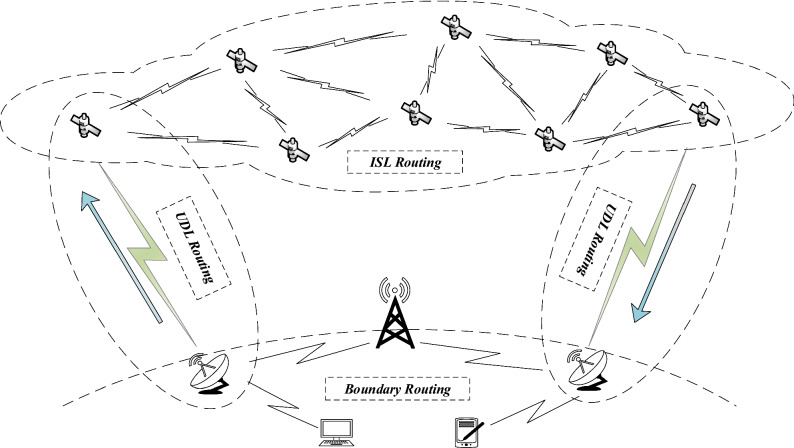
Under the perspective of routing domains, the routing of SINs can be divided into three types: UP and Down Links (UDLs) routing, boundary routing, and Inter-Satellite Links (ISLs) routing .

Under the perspective of routing domains, the routing of SINs can be divided into three types^13^: UP and Down Links (UDLs) routing, boundary routing, and Inter-Satellite Links (ISLs) routing in Fig. 2.

- **UP and Down Links Routing.** UP and Down Link routing links the satellite entities and the ground gateway or mobile user^14^. It is responsible for controlling various users/entities to the SINs and selecting the source entities and the destination entities.
- **Boundary Routing.** The fundamental function of border routing is to exchange network accessibility information about other autonomous systems (AS), which contains a list of AS to pass through to a destination. It is not the same as the ground network. The boundary routing in the SINs runs in border gateways and earth stations. Its function is to make the space-based network or the terrestrial network seamlessly interoperates and links so that users communicate with the space-based system by the terrestrial network transparently.
- **Inter-Satellite Links Routing.** In SINs, any satellites connected within the line of sight via ISLs^7^. ISL’s role is in the internal constellation network between the source and destination calculation of the optimal path to satisfy individual requirements. There are two kinds of ISLs: the links between neighboring satellites in the same orbital plane, intra-plane ISLs. And the connections between adjacent satellites in different orbital planes, inter-plane ISLs. A satellite entity could maintain 4-8 ISLs sometimes^15^. In general, the LEO satellite network routing part of the SINs refers to ISLs routing without special instructions.

At present, most of the SINs routing research focuses on ISLs routing of LEO satellite networks, which rend in two: connection-oriented and connectionless satellite network routing. Connection-oriented routing refers to ATM switching or ATM-like switching on the satellite, including DT-DVTR^16,17^, FHRP^18–20^, PRP protocols^19^, etc. Connectionless routing means the distributed forwarding packets on the satellite, including the Darting algorithm^21^, DRA algorithm^22^, etc. So the following only briefly introduces the connection-oriented routing algorithm and reviews the connectionless routing algorithm in detail. There are secure routing techniques involved regardless of how many layers there are, both single-layer and multi-layer.

### Single-layer SINs routing technology

There have intensive studies on satellite routing protocols in the literature, while not investigated on SINs routing. Therefore, this section reviews the single-layer SINs routing strategy, unless otherwise specified, which generally refers to the single-layer satellite network routing strategy.

The dynamic topology of SINs shows periodicity and predictability. Based on this feature, the routing technology first uses topology control strategies to shield the topology dynamics and then calculates routing on static topology sequences. In early times, the topology control strategy of SINs mainly includes the virtual topology strategy^22–24^, virtual node strategy^25,26^, and coverage domain separation method^27^. Later, researchers primarily focused on data-driven approaches^28–30^. So, single-layer satellite network routing algorithms can rend into four categories as following.

**Figure 3:**
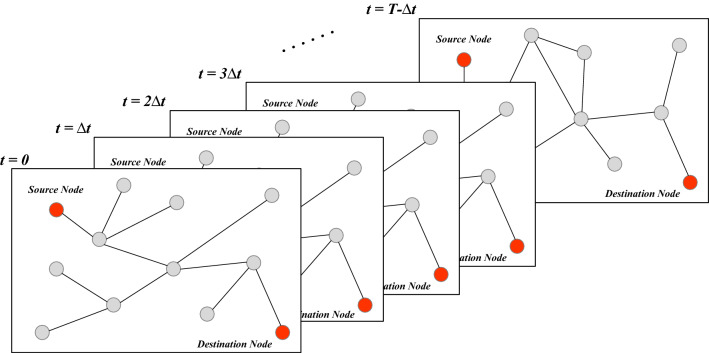
The virtual topology routing algorithm divides a period of the spatial information network *T* into a series of time segments $[t_0,t_1],[t_1,t_2],\ldots ,[t_{n-1},t_n]$. Within each time segment, the network topology and ISLs changes occur only at switch time point $t_1,t_2,...,t_n$. If the time segment $[t_i,t_j]$ is small enough, it uses the shortest path algorithm to find the optimal routing and back-up routing within this time segment.

- **Routing Algorithm Based on Virtual Topology.** The virtual topology routing algorithm divides a period of the SINs *T* into a series of time segments. Within each time segment $[t_0,t_1],[t_1,t_2],\ldots ,[t_{n-1},t_n]$, the network topology and ISLs changes occur only at switch time points $t_1,t_2,...,t_n$. If the time segment $[t_i,t_j]$ is small enough, it uses the shortest path algorithm to find the optimal routing and back-up routing within this time segment, as shown in Fig. 3. DT-DVTR (Discrete-Time Dynamic Virtual Topology Routing)^16,17^ is a connection-oriented routing algorithm based on the ATM. It is also the first satellite network routing algorithm based on virtual topology. DT-DVTR has two stages, Discrete-Time Virtual Topology Setup (DT-VTS) and Discrete-Time Path Sequence Selection (DT-PSS). Like the DT-DVTR algorithm, the Finite State Automata Route (FSA) algorithm^31^ also splits the dynamics period into finite time segments and is also a connection-oriented routing algorithm. But the difference is that FSA regards the network topology corresponding to each time segment as a state, which means that it abstracts the SINs as a Finite State Machine (FSM). These states depend on the ISLs connection. Snapshot-based routing is also a connection-oriented routing algorithm. Fischer et al.^32^ described a formal definition of the snapshot, and Gounder et al.^24^ defined a snapshot of SINs as the network topology at a specific moment. It created a new snapshot when adding new ISLs or disconnecting existing ISLs. Compact Explicit Multi-Path Routing (CEMR) algorithm^33^ is an effective adaptive routing for SINs. The basic idea is to encode the path (e.g., using the path identifier PathID). In this way, the intermediate nodes on the trail directly forward packets according to the information described by the PathID. The Explicit Load Balancing (ELB) algorithm^34^ achieves the load balancing of the network and avoids data packet loss as much as possible. The priority-based Adaptive Routing (PAR) algorithm^35^ proposed a priority-based adaptive shortest path routing policy. As a result, it is possible to effectively use ISLs, traffic in the network distributed evenly, improving system performance.
- **Routing Algorithm Based on Virtual Node.** Mauger et al.^25^ were the first to suggest virtual nodes, which were eventually used by Ekici et al.^26^ as part of the LEO satellite network’s distributed routing system. T.H.Chan et al. modeled the polar orbit constellation into a regular MSN and proposed Localized Zone Distribution Routing (LZDR)^36^. LZDR does not divide the routing domain according to a single virtual node but combines several adjacent virtual nodes into a zone and divides the routing calculation process into intra-domain routing and inter-domain routing. Datagram routing algorithm (DRA)^37^ is a datagram routing algorithm applicable to LEO satellite networks. The DRA algorithm introduces the virtual node concept, which generates a minimum propagation delay path between the source and the destination. The trail created by DRA has no loop, and the datagram determines the route autonomously. The protocol can still route datagram in case of a partial node failure, but its reduced performance.
- **Routing Algorithm Based on Coverage Domain Separation.** The optimization goal of Probabilistic Routing Protocol (PRP)^19^ is to reduce re-routing. The network establishes links between nodes according to the probability distribution function. When $P(min(T_c,T_hr)<T_{i,lh})$, the link terminates. $T_c$ means calling duration, $T_hr$ means times to recalculate the route after switching nodes, $T_{i,lh}$ means link switching time. However, PRP will increase the network constrictive, so it needs to balance re-routing and constrictive. Distributed Geographic Routing Algorithm (DGRA)^38^ is a hybrid method of geographic information routing and shortest path routing within a restricted zone. By geographic address, a simple distributed routing protocol indicates entity forwards the data packet to which direction. Use the shortest path algorithm to deliver packets within the limited destination’s restricted range when they arrive at the destination. SIPR framework extends the IP method to SINs, a satellite network routing algorithm based on IP. Hashimoto et al.^27^ first proposed an IP-based routing algorithm for LEO satellite networks. However, in the literature^39–41^, researchers have also offered some other distributed routing algorithms based on IP, and the primary routing strategy is still the same as DRA. In addition to significantly reducing the routing calculation and storage load, these distributed algorithms can eliminate the effects of satellite failure and traffic congestion to a certain extent. Still, their routing decisions are limited to local information and cannot solve these problems globally.
- **Routing Algorithm Based on Data-Driven.** Darting algorithm is the earliest connectionless SINs routing algorithm. It uses two data-driven routing update mechanisms: predecessor update and successor update^21^. The two mechanisms are responsible for updating the topology view on the next-hop node, which the data packet will arrive, and the topology view on the current node’s predecessor. SINs have many similarities with the Ad Hoc network on the ground, such as entities mobile, hybrid communication, and so on. Location-Assisted On-demand Routing (LAOR) algorithm^42^ introduces the On-Demand routing idea of Ad hoc network into SINs to form a new routing strategy. Zhou et al. pointed out that the LAOR protocol is not designed with security measures such as user terminal authentication and network node entity authentication, making the routing process vulnerable to attacks such as black hole attacks, impersonation, DOS, Byzantine attacks, and route replay attacks. They proposed a secure routing protocol based on LAOR protocol with an identity-based cryptographic regime (S-LAOR^43^). This scheme guarantees the integrity and confidentiality of routing information and the safe operation of routing protocols by introducing an identity-based cryptographic method to encrypt and authenticate the routing control packets in the network.

### Multi-layer SINs routing technology

The previous section introduces routing algorithms, which are only suitable for LEO single-layer satellite networks. However, a multi-layer network consisting of Low Earth Orbit (LEO), Medium Earth Orbit (MEO), and Geostationary Earth Orbit (GEO) has better performance than a single-layer network. Therefore, this section reviews various SINs routing algorithms for the multi-layer network, including MLSR^44,45^, SGRP^46^, HSRP^47^, and Agent-based algorithms^48^.

**Figure 4:**
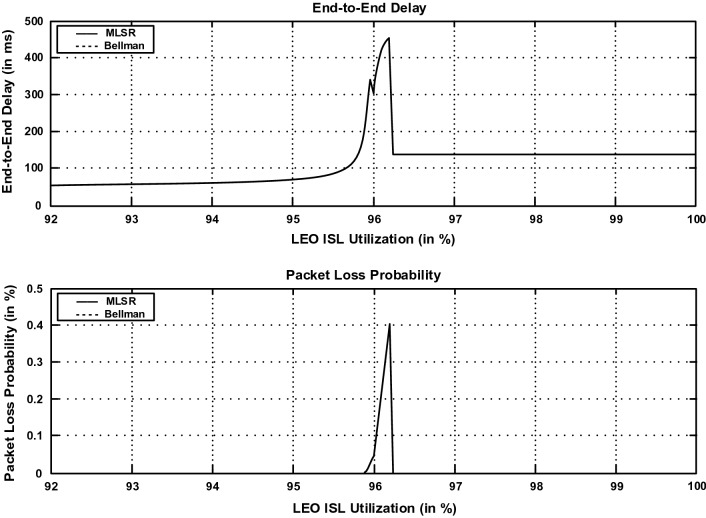
Delay and packet loss probability performance of the MLSR and shortest path routing algorithms. Because the performance of MLSR has the same performance as the shortest path algorithm, consequently, both curves are overlapped.

**Figure 5:**
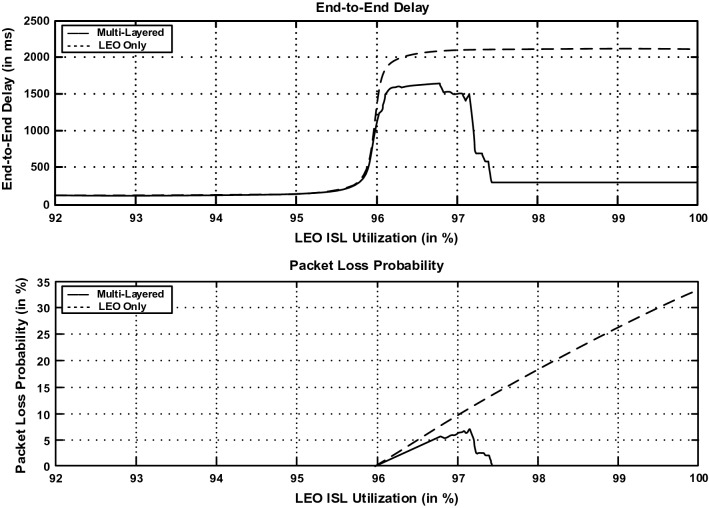
Delay and packet loss probability performance of multi-layered satellite network and an LEO satellite network.

Multi-Layered Satellite Routing (MLSR) Algorithm can efficiently calculate the shortest paths between different satellites in the satellite network and various entities in the terrestrial network. MLSR updates the routing table regularly to reflect changes in entity movement and network topology. In Fig. 4, the delay performance and the packet loss probability of the MLSR algorithm and the Bellman’s shortest path routing algorithm are depicted, and demonstrates that MLSR performs similarly to the shortest path algorithm in the single-layer network. Consequently, both curves are overlapped. Except for a slight swing when the route leaps to a higher entity, which is called the oscillatory phase. The end-to-end delay during the oscillatory phase is greater than the level attained after this phase ends, stabilizing around 130 ms. In addition, the likelihood of packet loss increases during this phase. Outside of the oscillatory zone, the packet loss probability is considerably smaller (very near to zero), and this is not visible in Fig. 4. Figure 5 demonstrated the routing performance gain obtained by using the MLSR in multi-layer satellite network architecture. For the single-layer (LEO) satellite network, packets are routed using routing tables created by the Bellman’s shortest path algorithm. The single-layer routing technology provides much worse end-to-end delays and loss probabilities when the LEO ISL utilization goes into the oscillatory region. MLSR performs better than single-layer network routing when the network load is high. The end-to-end delay performance of the LEO satellite network increases up to 2100 ms, and drops down to 350 ms after the oscillatory phase of the multilayered satellite network. In the LEO satellite network, the packet loss probability increases up to 34%, whereas the loss probability is always below 6% in the multilayered satellite network. Besides, MLSR divides routing calculations into multiple entities, and there is less computation overhead of routing tables.

Satellite Grouping and Routing Protocol (SGRP)^46^ is a double-layer network routing algorithm consisting of LEO and MEO. The main idea of SGRP is to transmit packets on the path with minimum delay and assign the routing table of the LEO entity to the MEO entity. MEO’s administrator calculates the minimum delay routing for the LEO entity based on LEO’s propagation delay report. Simulation results show that the end-to-end delay of SGRP does not increase with the data transmission rate, which is not different from the shortest path algorithm (e.g., Bellman). The performance of SGRP is close to Bellman in the case of forwarding node failure and link congestion. TARP-HL^49^ ensures that routing protocols work properly by signing, authenticating, and integrity verifying routing control information using a lightweight signature technique based on elliptic curves Pintsov-Vanstone Signature Scheme (ECPVSS). The protocol is easily adaptable to SINs and offers good security with little computational cost.

Hierarchical Satellite Routing Protocol (HSRP)^47^ offers a multi-layer, hierarchical network topology called Satellite Over Satellite (SOS) to meet the demands of broadband data transmission and multimedia services while minimizing data transmission latency with SINs. HSRP is a multi-layer network routing algorithm applied to SOS. On this basis, YU et al.^50^ proposed a secure routing protocol (TSRP) for multi-layer satellite networks, which effectively achieves trusted mutual authentication between source and destination nodes by using an authentication system to resist DoS attacks. The machine uses digital encryption technology for transmission security. It uses signature and timestamp techniques to ensure end-to-end data security and reliable transmission while protecting SINs from replay and black hole attacks.

The double-layer dynamic routing algorithm based on mobile-agent is a new novel routing algorithm^48^. It considers the essential characteristics of LEO/MEO and frequent link switching. It has a successful distance-vector experience on terrestrial routing, uses the logical terrestrial address as the basis for forwarding data packets, and uses the mobile agent’s autonomy and mobility to retrieve routing information. Zhang et al.^51^ proposed a mobile agent-based dynamic secure routing protocol. This system uses a reputation-based security strategy to filter out untrustworthy satellite nodes from routing decisions and steer traffic away from them. This method also includes encryption techniques to ensure that routing control information is transmitted securely.

### Intelligent routing based on deep learning

The rapid development of SINs assesses many new applications. These applications require various service quality types, which is a significant challenge towards the current best-effort routing algorithms of the terrestrial network. Since the recent extremely successful work using machine learning in computer vision, natural language processing, autonomous vehicles, and other fields. Many literature works^52–55^ try to design a ”smart” routing algorithm based on machine learning or deep learning methods.

**Figure 6:**
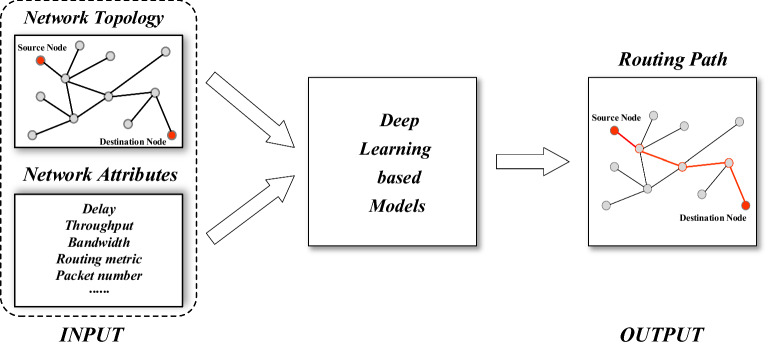
Scheme of deep learning based routing model.

Machine learning-based routing algorithms, in contrast to classic mathematical model-based, distributed routing algorithms, are generally data-driven and can adapt to dynamically changing network environments of SINs and meet varied service quality needs of the user. The most direct application of deep learning in routing optimization problems is to use deep learning models to replace the original routing algorithm based on mathematical models. Figure 6 shows a general intelligent routing solution model using a deep learning model.

**Figure 7:**
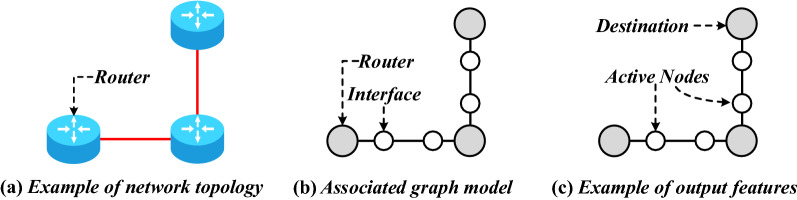
(**a**) Example network topology. (**b**) Its associatedgraph used for training. (**c**) The output feature of the inter-faces according to a selected destination^56^ .

- **Intelligent Routing Algorithm Based on Reinforcement Learning.** As early as 1993, Boyan et al.^57^ proposed Q-routing, an intelligent routing algorithm based on Q-Learning and applied in communication networks. In contrary to a traditional shortest-path algorithm, Q-routing can avoid network congestion and reduce transmission delay. Hu et al.^58^ proposed the QELAR method, which applied the idea of Q-Learning to wireless sensor networks (WSN) to optimize the energy consumption and lifespan of WSN. After that, related works^59–62^ improved and optimized this method. Due to various reasons, it is a challenging job to deploy Q-routing in real network scenarios.
- **Intelligent Routing Algorithm Based on Graph Neural Network.** The network’s local or global topology information is essential for completing intelligent routing decisions. However, due to the dynamic changes of SINs topology, traditional machine learning or deep learning models are often difficult to handle this part of information well. Graph neural network (GNN)^63^, proposed in recent years, is considered a new type of neural network structure that can effectively deal with topological information extraction problems. Geyer et al.^56^ designed a distributed intelligent routing algorithm based on GRU and GNN. This method adds the router interface as extra nodes to the graph model to help the GNN model express the routing network structure’s characteristics better and make the network feature information described by the GNN easier for the routing decision process. Figure 7 shows the schematic diagram of the graph model with additional nodes for the router interface. The work of Rusek et al.^64^ combined GNN with the LSTM model. It used a deep learning model based on Graph Neural Network to model the relationship between routing path delay, delay jitter, network topology, traffic matrix, and routing path. The established model is used to assist the heuristic routing optimization algorithm in calculating the routing strategy.

In addition to the rapid development of artificial intelligence technology, Software Defined Network (SDN)^65^ and programmable routing equipment^66,67^ that have emerged in recent years also provide new ideas for intelligent routing algorithms. To characterize the features of different routing technologies in SINs, we analyzed and compared them from nine perspectives, as indicated in Table 3: connection method, policy, tolerance of failure, delay, load, convergence, overhead, computation mode, and training mode.

**Table 3:** Comparison of routing technologies in SINs.

Routing technology	Connection method	Optimization objective/routing policy	Tolerance of failure	Delay	Load	Convergence	Overhead	Computation mode	Training mode
DT-DVTR^16,17^	Connection- oriented	1.QoS 2.Minimum handovers	Low	Normal	**–**	Low	Low	Centralized	Offline
FSA^31^	Connection- oriented	1.QoS 2.Minimum handovers	Low	Low	Imbalance	Low	Low	Centralized	Offline
CEMR^33^	Connectionless	Traffic balance	Normal	Low	Balance	Normal	Normal	Distributed	Online
ELB^34^	Connectionless	Congestion avoidance	Low	Normal	Balance	Normal	High	Distributed	Online
PAR^35^	Connectionless	Traffic balance	Normal	Low	Balance	Normal	Low	Distributed	Online
LZDR^36^	Connection- oriented	Minimum hop count	Low	**-**	Imbalance	High	Normal	Centralized	**-**
DRA^37^	Connectionless	Minimum propagation delay	Normal	Low	Balance	Low	Low	Distributed	Online
PRP^19^	Connection- oriented	Minimum Re-routing	Low	High	**-**	High	High	Centralized	Online
DGRA^38^	Connectionless	Embed Geographic Information	Low	Normal	**-**	Normal	High	Distributed	Online
SIPR^27^	Connectionless	Minimum end-to-end delay	Normal	Low	**-**	Low	Normal	Distributed	Online
Darting^21^	Connectionless	Minimum Topology Update	Normal	Normal	Balance	Low	High	Distributed	**-**
LAOR^42^	Connectionless	1.QoS 2.Minimum end-to-end delay	High	Low	Balance	Low	Low	Distributed	**-**
MLSR^44^	Connection- oriented	Minimum end-to-end delay	Normal	Low	Balance	Low	Normal	Centralized	Online
SGRP^46^	Both	Minimum end-to-end delay	High	Low	**-**	Low	Low	**-**	**-**
HSRP^47^	**-**	QoS	**-**	Low	**-**	Normal	**-**	**-**	Online
Agent-based^48^	**-**	Distance vector	Normal	Normal	Balance	Normal	Normal	**-**	Online
QELAR^58^	Connectionless	Path generation	High	Low	Balance	Low	High	Distributed	Online
Q-routing^59^	Connection- oriented	Adaptive	High	Low	Balance	Low	High	Centralized	Offline
GNN and GRU based^56^	Connectionless	Path generation	Normal	Low	Balance	Low	Low	Distributed	Offline
GNN and LSTM based^64^	Connection- oriented	Delay andjitter prediction	High	Normal	Balance	Normal	Low	Centralized	Offline

## Anomaly detection in SINs

The term anomaly detection, also known as outlier analysis, in networks refers to the problem of finding exceptional patterns in network that do not conform to the expected normal behavior^68^. Anomaly detection is a fundamental problem with multiple applications, and various research domains have studied it for decades. Because the SINs is also a dynamic network^69^, anomaly detection in SINs could draw on the dynamic network anomaly detection method’s experience. At the end of this section, we show an anomaly detection scheme for SINs based on graph embedding.

### Anomaly detection in dynamic network

In the past, network science research focused on static networks, which the links are fixed between nods. However, as research continues to deepen, people have discovered that most natural and social systems are dynamic networks, which, unlike static networks, are constantly changing to their structure or attributes^70^. Social networks, citation networks, electric power grids, global financial systems, and SINs mentioned in this paper are examples of dynamic networks. Insertion and deletion of vertices(objects), insertion and deletion of edges (relationships), and modification of features (e.g., vertex or edge labels)^71^ are all examples of dynamic changes.

In these networks that change at any time, some elements show abnormal behaviors, whose change rules or characteristics are different from other elements. Such scenarios include: disseminating false information in social networks, the abrupt suspension of academic cooperation in citation networks, the surge in electricity consumption at a specific node in electric power grids, the massive flow of funds in global financial systems, the node under threat in SINs, so forth.

There are four different types of anomalies: vertices, edge, subgraphs, and event.

- **Anomalous vertices.** Anomalous vertex means a subset of the vertices such that every vertex in the subset has an *”active”* or *”irregular”* evolution compared to the other vertices in the graph.
- **Anomalous edge.** Like anomalous vertex, anomalous edge means finding a subset of the edges such that each edge in the subset has an *”active”* or *”irregular”* evolution, optionally identifying the time points where they are abnormal.
- **Anomalous subgraphs.** Anomalies of this type, unique to dynamic networks, including communities that split, merge, disappear, and reappear frequently or exhibit some other behaviors. Anomalous subgraphs in the network usually appear in subgraphs’ form, such as congestion zones in traffic networks and hot groups in social networks.
- **Anomalous event and change.** Unlike the previous discussed, this anomalous are found only in dynamic graphs. Events occurred at an isolated point in time when the graph differs from the graphs at prior and subsequent time points. If the time point is isolated, the surrounding time points are very dissimilar, indicating an anomalous event occurred. Besides, the persistence of the new edges suggests that a change, not an event.

An essential problem over dynamic networks is anomaly detection—finding different objects, relationships, or points in time. However, how to mine oddball elements in dynamic networks is a more complex problem. Initially, techniques focused on anomaly detection in static graphs, mainly focusing on the network’s structural characteristics and detecting the abnormal elements by looking for the strange changes in the structure. It is worth noting, however, that dynamic networks contain attribute qualities in addition to structural properties. As a result, the anomaly detection issue for dynamic network data must take into account the structure and characteristics of the graph’s members at the same time.

Aggarwal et al.^72^ paid attention to anomaly detection in graph streams. They believed that the anomaly in graph streams is edging connecting different tight regions and then proposed modeling the edges in a dynamic network with a structural connectivity model. Ranshous et al.^73^ focused on edge anomaly detection and modeled the dynamic network as edges that changed over time, and based on this, detected irregular edges. They set up three empirical anomaly indicators - sample score, preferential attachment score, and homophily score - to score the change edges to define anomaly. Manzoor et al.^74^ focused on anomaly detection in heterogeneous graphs. Heterogeneous graphs are graphs in which points (edges) can have multiple forms, such as knowledge graphs. Yu et al.^75^ first applied deep learning technology to dynamic network anomaly detection. After that, Guo et al.^76^ proposed a network anomaly detection algorithm based on Graph Neural Network that can capture the nodes and edges’ attributes and time-varying features and fully uses them to learn a representation vector for each node. This method can capture both structural and attribute anomalies. Ranshous et al.^71^ constructed a two-tiered taxonomy. They first partitioned the methods based on community, compression, decomposition, distance, and probabilistic model. Then, subdividing anomaly detection methods based on the categories of abnormalities detected.

### Anomaly detection methods in SINs

Due to the high maintenance and operating cost and potential risks in the space environment, accurately detecting anomalies in SINs is crucial yet challenging for several reasons. SINs is susceptible to a broad spectrum of strange behavior, which might be caused by irresistible natural forces or malevolent man-made attacks. It may even be complex system interactions from various internal and external factors. The detection of anomalies in these systems is a complex problem and made more difficult by the unique nature of SINs^77,78^.

Most of the previous works^79–81^ on detecting anomalies in satellite systems have primarily focused on simple threshold techniques of single parameter measurements to alert the operator to unusual events. Sylvain et al.^79^introduce an innovative anomaly detection method based on machine-learning algorithms. This method can detect abnormal behaviors that were not immediately detected by ordinary monitoring system. However, the false alarms rate of this method is relatively high. Kyle et al.^80^demonstrate the viability of LSTMs for predicting spacecraft telemetry and propose a novel dynamic thresholding approach that does not rely on scarce labels or false parametric assumptions. Corey et al.^81^introduced GSOCs approach to automated telemetry monitoring as the Automated Telemetry Health Monitoring System (ATHMoS). ATHMoS uses supervised learning methods to attempt to solve the anomaly detection problem by comparing new telemetry data with past nominal and anomaly data. However, those approaches fail to detect more nuance anomalies, which occur when several variables present in a rare combination. Therefore, Arbon et al.^82^ described how to trained a One Class Support Vector Machine (OCSVM) to detect anomalies using historical multivariate data from a satellite communications network. The trained OCSVM examines multiple variables simultaneously, rather than looking at each in isolation, and is able to perform real-time outlier detection and quantify how abnormal the outliers are. The advantage of the OCSVM is that the training data does not need to include anomalies, nor is the labelling of ’normal’ versus ’anomalous’ data required. Assume the challenge of finding outliers in high-dimensional time-series data, such as transponder frequency spectra, has been accepted. It can use recurrent neural networks, particularly Long Short Term Memory (LSTM) networks, to detect anomalies^83–85^. Gunn et al.^86^ consider the problem of detecting outliers in high-dimensional time-series data and using an LSTM network for anomaly detection. This approach significantly improves on simple threshold models and moving average and static predictors. Although many literature studies have studied rule-based or machine learning-based anomaly detection approaches, they have not explored the tensor-based decomposition method extensively. Shin et al.^87^ proposed an Integrative Tensor-based Anomaly Detection framework (ITAD) to reduce false positives in the KOMPSAT-2 satellite telemetry dataset by analyzing multiple telemetries simultaneously, thus incorporating information from multiple telemetries at the same time. ITAD achieves higher performance in terms of precision and F1 score compared to other approaches. ITAD significantly reduces the number of false positives by correctly identifying actual anomalies and trivial outliers.

Due to this paper’s space limitation, it is impossible to list all anomaly detection methods, so only a few well-known techniques as an example are compared. Table 4 shows the outcomes of the comparison. We compare situations, anomalous objects, benefits, drawbacks, evaluations in the datasets. SINs are the most common detection situations in methods^79–82,86,87^. Except for OCSVM, the other techniques’ detection objects are vertices, edges, and events. The primary differences in detection time and detection effectiveness are the advantages and disadvantages of various detection approaches. Both remote sensing and sensor data are included in the dataset. Different methods or data sets correspond to different evaluation scores. The performance metrics this survey collected to tune the detection algorithm parameters include the number of AUCs, False Positive Rate (FPR), accuracy, and precision. Different parameters and experimental environments yielded different experimental results. The highest AUCs are close to 1.0 from the StreamSpot method. FPR of GOutlier method on R-mat < 0.2. Most of the methods use accuracy as an evaluation metric, and therefore it has the largest variance. The highest accuracy is even higher than 0.95. Precision is ideal. Only SpotLight’s precision is between 0.57 and 0.96.

**Table 4:** Comparison of anomaly detection methods in SINs.

Method	Scenario	Anomalous object	Advantage/disadvantage	Dataset	AUCs	FPR	Accuracy	Precision
GOutlier^72^	Graph streams	Vertices	Processing rate can vary somewhat over time.	DBLP, IMDB, R-Mat	–	R-Mat: $\leqslant 0.2$	–	–
Method^73^	Graph streams	Edges	Achieve high precision on identifying injected outliers.	DBLP, RTG	Both around 0.97	–	–	–
StreamSpot^74^	HeterogeneousGraphs	Edges	Scoring and flagging anomalies quickly in real time.	YDC, GFC, ALL	Close to 1.0	–	> 0.95	> 0.90
Netwalk^75^	EvolutionaryNetwork	Vertices, edges	Flexible to be applied on different types of networks.	UCI Messages,arXiv hepth,Digg, DBLP	UCI: 0.7226–0.7758,arXiv: 0.6939–0.7489,Digg: 0.6837–0.7563,DBLP: 0.74–0.80	–	UCI: 0.73–0.76,arXiv: 0.73–0.77,Digg: 0.71–0.80,DBLP: 0.6858–0.7654	–
Dynamic-DGI^76^	Temporal Networks	Subgraphs	Graph structure, edges’ attributesand time-varying featuresare introduced into the model.	IDS2017, Digg,Reddit Hyperlink Network	–	–	IDS 2017:0.91±0.02Digg:0.81±0.01	–
SpotLight^88^	Graph Streams	Subgraphs	Fast speed and high accuracy.	DARPA, ENRON,NYCTAXI	< 0.9	–	–	0.57–0.96
Method^79–81^	SINs	Vertices, edges, events	Fail to detect more nuance anomalies.	HKTM$^{1}$, ISAs$^{2}$	–	–	HKTM: 0.875–0.99	ISAs: 0.875
OCSVM^82^	SINs	Events	More sensitivity and specificity.	[c]Spectral datafrom receiver	–	–	0.9499	–
Method^86^	SINs	Vertices, edges, events	Improves on simple thresholdmodels, moving average and static predictors.	Spectral datafrom receiver	–	–	0.88–0.92	–
ITAD^87^	SINs	Vertices, edges, events	Achieving higher accuracy and lower false positive rates.	KOMPSAT–2$^{3}$	–	$\approx 0.3333$	–	> 0.9

$^1$The HKTM (House Keeping TeleMetry) parameters recorded permanently by a spacecraft in order to monitor its health.

$^2$A subset of all of the incidents and anomalies detailed in ISAs manifest in specific telemetry channels. This dataset collect by expert-labeled telemetry anomaly data from the Soil Moisture Active Passive (SMAP) satellite and the Mars Science Laboratory (MSL) rover, Curiosity.

$^3$KOMPSAT-2 collects more than 3,000 different types of telemetries data from Korea Multi-Purpose Satellite-2.

### A proposed anomaly detection schemes

The knowledge graph technology that has emerged in recent years has widely been used in semantic search^89^, intelligent question & answer systems^90^, personalized recommendations^91^, and decision-making assistance^92^. In the field of security, the application of a knowledge graph is still in the exploratory stage. However, the graph model has been applied in many security field scenarios and has achieved good results. The cybersecurity knowledge graph incorporates more knowledge into the original graph model, providing more semantic information for detection, analysis, and response to anomalies.

**Figure 8:**
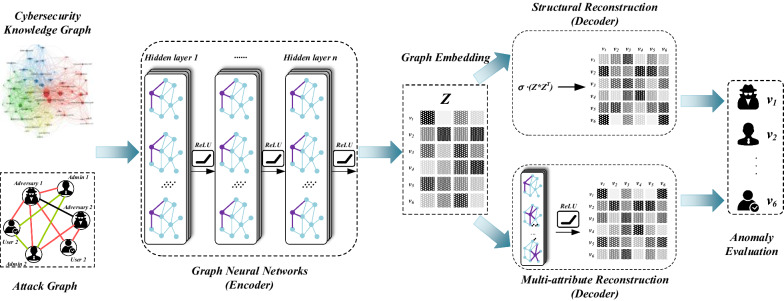
This paper propose an anomaly detection scheme based on the cybersecurity knowledge graph. The framework mainly includes three parts: construction of graph model, graph embedding, and anomaly evaluation. First, taking the cybersecurity knowledge graph and attack graph as input, the graph neural networks (encoder) embedding the graph’s vertices to obtain the attack graph’s characteristic representation. Then add structural anomaly features and attribute anomaly features, graph neural networks (decoder) learn the threat degree of each node to other nodes, and aggregate the node’s threat degree. After many iterations, the model finally gets each node’s anomaly ranking in the cybersecurity knowledge graph.

Based on the above discussion, this section summarizes and designs an anomaly detection scheme based on the cybersecurity knowledge graph, as shown in Fig. 8. The framework mainly includes three parts: construction of graph model, graph embedding, and anomaly evaluation.

First, the graph neural networks (encoder) embed the graph’s vertices to generate the attack graph’s characteristic representation, using the cybersecurity knowledge graph and attack graph as input. Then add structural anomaly features and attribute anomaly features, graph neural networks (decoder) learn the threat degree of each node to other nodes, and aggregate the node’s threat degree. After many iterations, the model finally gets each node’s anomaly ranking in the cybersecurity knowledge graph. The whole process can be represented formally as follows:

$$\begin{aligned} min\ {\mathbb {R}}\left[ dist(\mathbf{X },\ Dec(Enc(\mathbf{X }))) \right] \end{aligned}$$

where the graph’s adjacency and attribute matrices are denoted by $\mathbf{X }$, the depth encoder is Enc(), the decoder is Dec(), and the distance function is dist(), the anomaly node ranking is ${\mathbb {R}}$.

The graph model construction stage determines the entities and relationships in the graph and forms a network cybersecurity knowledge graph. There are two types of cybersecurity knowledge graphs: static and dynamic. Static knowledge graphs are security knowledge graphs that are built ahead of time and include numerous knowledge repositories such as attack pattern repositories (APR), security risks (SR), malicious code (MC), and attack target assets (ATA). Those knowledge don’t have to be updated in real-time. Dynamic knowledge graphs is the real-time alarms generated by entities devices and some information related to the alarms, such as IP, port, network segment, alarm, file, log, and other entities. Through shared entities, dynamic and static knowledge graphs establish particular relationships. For example, IP addresses are associated with ATA. Alert messages are associated with ARP, MP, ATA and so on. However, the relationships include explicit relationships and implicit relationships. The explicit relationship is a direct relationship, while the implicit relationship requires data mining methods to obtain the relationship between data. This essay will use IP as an example for the sake of convenience. An alarm sequence is created after aggregating alarms based on the source and target IP within a unit time window. The starting sequence vector of the current alarm sequence may be determined by iterating over the alarm sequences of all IP pairs using the following formula, assuming that the current alarm sequence is *s*, the probability of observing an alarm sequence *w* at time *t* is *Pr*(*w*).

$$\begin{aligned} {\tilde{s}} \propto \frac{1}{\left| s \right| }\sum _{w \in s} (\frac{a}{a+Pr(w)}\cdot v_{w}) \end{aligned}$$

where $a= \frac{1-\alpha }{\alpha Z}$, $\alpha$ is the hyperparameter and *Z* is the normalization result, the vector of alert sequences *w* is $v_{w}$.

The graph embedding stage analyzes the constructed cybersecurity knowledge graph and uses graph embedding to map different anomaly detection dimensions to the same space. The encoding of the cybersecurity knowledge graph must take into account not only the encoding of the graph structure but also the encoding of the node attribute. The encoder utilizes a graph convolutional neural network, which formal specification by the formula following.

$$\begin{aligned} H^{(l)}=\, & {} f(H^{(l)}, X\mid W^{(l)})\\f(H^{(l)}, X\mid W^{(l)})=\, & {}  \sigma \left( {\tilde{D}}^{-\frac{1}{2}}{\tilde{X}}{\tilde{D}}^{-\frac{1}{2}} H^{(l)} W^{(l)}\right) \end{aligned}$$

where *X* means graph structure *S* or node attribute *A*.

In the anomaly evaluation stage, the structure reconstruction decoder reconstructs the network topology by embedding nodes. The attribute reconstruction decoder reconstructs the attributes of nodes in the attribute graph through the embedding of nodes. The decoding process is to predict the existence of edges between entity pairs using the encoder’s feature representation *Z* as input, similar to link prediction. Respectively, the formal specification for the decoders of structural and multi-attribute reconstruction are:

$$\begin{aligned} {\hat{S}}=\, & {} \sigma \left( Z\star Z^{T} \right) = sigmoid\left( z_{i}, z_{j}^{T} \right) \\ {\hat{A}}=\, & {} f_{Relu}\left( Z, A\mid W^{(n)} \right) \end{aligned}$$

After a certain number of iterations, the anomaly score is calculated. The cybersecurity knowledge graph structure’s D-value of encoding and decoding measures the outliers of entities in the graph.

$$\begin{aligned} D\_{}value(e_{i})=(1-\alpha )\cdot \left\| S-{\hat{S}}_{i} \right\| _{F}^{2}+\alpha \cdot \left\| A-{\hat{A}}_{i} \right\| _{F}^{2} \end{aligned}$$

The graph convolutional network’s time complexity increases linearly with the number of entities and edges in the network. As a result, the method’s time complexity is $O\left( mdH + n^{2} \right)$, *m* represents the number of non-zero elements of the graph’s attribute matrix, *d* means the dimensionality of the features of the attribute matrix, *H* denotes the sum of the number of features of each graph convolution layer, and *n* indicates the number of nodes in the graph. With the more influence of features and anomalies, this scheme significantly improved the anomaly detection of different entities in the same spatial, both efficiency and accuracy compared with the traditional method. Theoretically, graph neural networks could carry out a unified representation of the cybersecurity knowledge graph’s entities and relationships. Thus, the representation of entities can get used for anomaly assessment, and the representation of relationships can get used for tracing attacks and predicting attacks. However, in reality, the SINs environment is more complicated, and it is difficult to find an effective embedding method to meet this demand, but this is what we are exploring.

## Future directions

The security technology study of SINs has always been a challenging problem due to the contradictions between changing network structure, growing user demand, and inadequate onboard computer capacity. So far, it proposes many security protection technologies, but there are still some key issues that need to be studied urgently.

### Uniform SINs security architecture and model

SINs will become a significant asset of 6G as a new product of the era and will thrive soon. Because of this considerable growth potential, individuals must pay close attention to the security architecture of SINs. Without a uniform security architecture and model, a robust network protection mechanism will be lost, causing significant damage to SINs. The existing security model does not suitable for describing the security risks for SINs. Therefore, SINs need a formal security model by considering the functionalities, characteristics, and security requirements of SINs. Because there is no standard definition describing the security features and security requirements for SINs, algorithm/protocol schemes are not concrete. The security of existing algorithms and protocols for SINs based on informally empirical analyses. They may have potential security loopholes when the natural environment deployed these algorithms and protocols. Although the security architecture of SINs is similar to the traditional network’s security architecture, there are apparent differences due to the characteristics of SINs, so it is necessary to design the network security architecture according to the new security requirements. A comprehensive security architecture should describe as follow. One is the potential hazard to SINs. The second need is for SINs to provide security services and procedures. The third category is security technologies, which should be defined in layers, as in the Open Systems Interconnection paradigm, to guarantee that SINs operate reliably. Fourth, network management features such as security situational awareness and security simulation verification are discussed.

### Secure space-air-ground computing architecture

With the global connectivity services/applications that demand continues to grow, such as ubiquitous communication, smart transportation, smart city, maritime surveillance, and disaster rescue, the terrestrial networks and computing alone cannot meet the needs effectively and efficiently. Therefore, space-air-ground edge computing will integrate satellite systems, aerial networks, terrestrial communications, and Cloud computing to become an emerging computing architecture and attract intensive research interest.

Mobile Edge Computing (MEC) is an open architecture and platform that can be deployed flexibly in different locations of SINs to meet various services and needs. For example, MEC can be deployed in the base station of the SINs access network to take up caching, task offloading and other functions with high time delay and computational requirements. MEC can also be deployed in satellite gateways to perform authentication, registration, QoS and other functions. The MEC server deployed at the edge of SINs’ subscriber network can perform the tasks of UPF (User Plane Function), such as billing, monitoring, and authentication. MEC in the SINs control and management unit enables mobility management, session management, billing management, and other functions. Services such as load balancing and VPN can be virtualized in the MEC virtual infrastructure at the terrestrial base stations, providing offloading computing resources and localized proprietary networks.

### Blockchain based application in SINs security

Blockchain uses a distributed network structure that has natural advantages in combination with satellites. Identification, self-reconfiguration, and decentralization are some of the possible blockchain applications in SINs security. Blockchain mainly includes the data, network, consensus, incentive, contract, and application layers.

The data layer is primarily a data ledger consisting of multiple transaction information and sub-block information, containing blocks for storing data, timestamps, asymmetric cryptography, and hash functions. Asymmetric key encryption might be used by SINs nodes to generate identities on a blockchain. Any modifications to data will have an impact on the blockchain as a whole, no guaranteeing the identity’s authenticity. The network layer is a distributed network with a peer-to-peer networking model. each node in the SINs can act as a routing node and deliver the received information. The control system of SINs introduces data consistency of blockchain in the consensus layer, which can design a spatially cooperative fault tolerance and intelligent reconfiguration mechanism. Those mechanisms can achieve efficient fault tolerance control and intelligent self-healing of the network and improve the overall resilience and robustness of the system. The incentive layer uses a specific mechanism to ensure that all nodes in the SINs are involved in the data block validation process. A data centre is established in orbit to provide global communications and secure in-orbit data storage to users. Blockchain on satellites eliminates dependence on ground-based infrastructure for data transfer, storage or computation, and eliminates significant vulnerabilities due to data leaks or data breaches. The contract layer includes data distribution, smart contracts and contract scripts. Blockchain could use smart contracts to improve the resilience of SINs. Attackers attempting to tamper with the control instructions of SINs could not modify the organization schemes of SINs contained in smart contracts, making them difficult to tamper with on a blockchain. Nodes at the same clusters could form a blockchain according to Spatio-temporal correlation, and this would achieve decentralization to some extent, reducing the evil influences of single-point fault.

In addition, there are cross-application scenarios between blockchain technology and SINs (even 6G), where blockchain provides trust mechanisms for the network and SINs offer connectivity services to the blockchain. The broadcast capability of SINs and quantum satellite teleportation will also play a unique role in the core blockchain functions. In short, blockchain-based SINs will be one of the most secure and trusted network systems available.

### Lightweight cryptographic algorithms and protocols

Secure data transmission and processing in SINs need to meet complex requirements. The basic idea is to decompose requirements based on data processing procedures. Then, one can propose corresponding algorithms, protocols, schemes to meet the different security requirements. Since entities in space suffer from the limitation of computation and communication capabilities, lightweight and fast encryption schemes provide the tradeoff between efficiency and security. AES (Advanced Encryption Standard) will be the effective encryption technique utilized in SIN to satisfy minimal security standards. In AES, there are five encryption modes: CTR (Counter Mode), ECB (Electronic CodeBook), CBC (Cipher-block chaining), CFB (Cipher Feedback), and OFB (Output Feedback). To encrypt data before transmission, CCSDS recommends utilizing the CTR mode in AES. Lightweight encryption methods, on the other hand, strive for the highest possible bit rate. It might reveal previously unknown security vulnerabilities in the planned schemes. If appropriate cryptographic methods with error correction and data authentication are developed, SINs will benefit significantly more.

When utilizing or developing new SINs strategies, there must be addressed the overheads of storage and computation. As a result, while choosing appropriate cryptographic algorithms and protocols or proposing new ones based on SINs, the following features should be considered.

### Available SINs security simulation and validation platform

Depending on the application scenario, several popular satellite Network simulation tools include Systems Tool Kit(STK), OPNET, Network Simulator(NS), and Mininet. STK and OPNET are commercial software, and NS and MININET are open-source research software. These are more focused on the simulation of network modeling, transmission protocols, routing algorithms, and no simulation function specifically for satellite network security. Furthermore, no simulation and validation platforms can verify the algorithms or protocols’ efficiency and safety. Therefore, it is necessary to comprehensively apply digital twin technology, cloud technology, network simulation technology, virtual reality technology, and human-computer interaction technology to build a virtual simulation platform specifically for SINs security.

This platform is different from the current isolated simulation and validation system, which includes verifying security technology on the SINs, the rehearsal of attack and defense, and the comprehensive judgment of SINs situation awareness. It consists of three parts: scenario and simulation, behavior modelling and simulation, security simulation and confrontation rehearsal. Each component meets the needs of each simulation task through a set of standard buses. The scenario modelling and simulation module uses STK to model network entities such as various nodes and links in SINs. The behaviour modelling and simulation module implements the simulation of DTN, CCSDS and other related protocols. It also needs to simulate various optimized feed-forward routing, link channel assignment optimization and service queue scheduling optimization mechanisms. The security simulation and confrontation Reasoning module constructs twin SINs through digital twin technology, accesses actual devices or simulators, and provides an environment for SINs attack and defence experiments. By using attack and defence control software to control the twin SINs nodes: for example, choosing to turn on the attack function, defence function and related parameter configuration of a node, and simulating the typical threats and attack and defence means facing the airspace information network, such as DDOS, wormhole, replay, intrusion detection, authentication, firewall, etc.

Overall, the security simulation and verification platform will serve as a simulation test platform for critical security technology research of SINs and a demonstration platform for verification, evaluation, and assisted decision-making of new spatial information systems.

## Conclusion

After the investigations, this paper found intensive studies on satellite networks and their security protocols. In contrast, it has not investigated SINs’ security issues well yet, let alone the related security issues combine with new emerging technology. Therefore, we study the security issues in SINs via surveying the existing work and future directions. Moreover, the secure and intelligent routing issues in SINs are also reviewed in four aspects, including SINs routing types, single-layer routing, multi-layer routing, and intelligent routing based machine learning. Besides, anomaly detection issues in SINs are investigated, and we proposed an anomaly detection scheme is presented. In conclusion, we hope that this paper gives an overview of the newly emerging technologies and provides some inspiration for future exploration concerning the security problems in SINs.

## References

[1] Hewu L (2016). Progress and tendency of space and earth integrated network. Sci. Technol. Rev..

[2] CCID Consulting IoT Industry Research Center. *”New Infrastructure” White Paper on the Development of China’s Satellite Internet Industry* (2020). https://n2.sinaimg.cn/tech/cbc3161f/20200528/SatelliteInternetWhitePaper.pdf.

[3] Yunhao, F. Russian hacker organization ”tula” hijacks satellite links to conduct cyber attacks. *Defense Viewpoint* (2015).

[4] Paganini, P. Hacking satellites ... look up to the sky. [EB/OL]. https://resources.infosecinstitute.com/topic/hacking-satellite-look-up-to-the-sky/. Accessed November 4, 2020.

[5] John, L. State cyberspies wriggle into satellites for super-duper sneaky ops. [EB/OL]. https://www.theregister.com/2015/09/09/turla_apt_satellite_stealth/. Accessed November 4, 2020.

[6] CNBC. China-based hacking campaign is said to have breached satellite, defense companies. [EB/OL]. https://www.cnbc.com/2018/06/19/china-based-hacking-breached-satellite-defense-companies-symantec.html. Accessed November 4, 2020.

[7] Jiang C, Wang X, Wang J, Chen HH, Ren Y (2015). Security in space information networks. IEEE Commun. Mag..

[8] Jianwei L, Weiran L, Qianhong W, DaWei L, Shigang C (2016). Survey on key security technologies for space information networks. J. Commun. Inf. Networks.

[9] Book, G. Security threats against space missions. *Inf. Rep.* (2006).

[10] He D, Li X, Chan S, Gao J, Guizani M (2019). Security analysis of a space-based wireless network. IEEE Network.

[11] ISO. Iso 7498-2:information processing systems—Open systems interconnection—Basic reference model—Part 2: Security architecture (1989).

[12] Yi, Z., Quan, Z., Jun, L. & Wei, L. The generation and update algorithm of routing table in satellite network. In *2015 IEEE International Conference on Communication Problem-Solving (ICCP)* (2015).

[13] Zaým, A. H. Routing in leo satellite networks: An implementation (2008).

[14] Boutin K, Lecours M, Pelletier M, Delisle GY (2010). Effects of fade distribution on a mobile satellite down-link and up-link performance in a frequency reuse cellular configuration. Int. J. Satellite Commun. Netw..

[15] A, J. Y., A, L. X., Pei, W. B., C, L. S. & A, Y. C. A scheduling strategy to inter-satellite links assignment in gnss. *Adv. Space Res.* (2020).

[16] Werner & M. A dynamic routing concept for atm-based satellite personal communication networks. *Sel. Areas Commun. IEEE J.* **15**, 1636–1648 (1997)

[17] Zhao, C. Y., Chen, Y. & Shao-Qian, L. I. Research on dt-dvtr based routing algorithm for compass satellite network. *Electron. Des. Eng.* (2018).

[18] Uzunalioglu, H. & Yen, W. Managing connection handover in satellite networks. In *In Proc. GLOBECOM ’97*, 1606–1610 (1997).

[19] Uzunalioglu, H. Probabilistic routing protocol for low earth orbit satellite networks. In *ICC ’98. 1998 IEEE International Conference on Communications. Conference Record. Affiliated with SUPERCOMM’98 (Cat. No.98CH36220)*, vol. 1, 89–93 vol.1 (1998).

[20] Uzunalio?Lu, H., Akyildiz, I. F., Yesha, Y. & Wei, Y. Footprint handover rerouting protocol for low earth orbit satellite networks. *Wirel. Netw.* **5**, 327–337 (1999).

[21] Kuang, T. & Ma, R. P. Darting: a cost-effective routing alternative for large space-based dynamic-topology networks. In *Military Communications Conference, 1995. MILCOM ’95, Conference Record, IEEE* (1995).

[22] Chen, C., Ekici, E. & Akyildiz, I. F. Satellite grouping and routing protocol for leo/meo satellite ip networks. In *in: 5th International Workshop on Wireless Mobile Multimedia (WoWMoM 2002*, 109–116 (2002).

[23] seong Chang, H. *et al.* Topological design and routing for low-earth orbit satellite networks (1995).

[24] Gounder, V. V., Prakash, R. & Abu-Amara, H. Routing in leo-based satellite networks. In *Wireless Communications and Systems, 2000. 1999 Emerging Technologies Symposium* (1999).

[25] Mauger R, Rosenberg C (1997). Qos guarantees for multimedia services on a tdma-based satellite network. Commun. Mag. IEEE.

[26] Ekici E, Akyildiz IF, Bender MD (2001). A distributed routing algorithm for datagram traffic in leo satellite networks. IEEE/ACM Trans. Netw..

[27] Hashimoto, Y. & Sarikaya, B. Design of ip-based routing in a leo satellite network. In *Proceedings of Third International Workshop on Satellite-Based Information Services (WOSBIS ‘98*, 81–88 (1998).

[28] Feng, X., Yang, M. & Guo, Q. A novel distributed routing algorithm based on data-driven in geo/leo hybrid satellite network. In *2015 International Conference on Wireless Communications & Signal Processing (WCSP)* (2015).

[29] Cao, J. Congestion control and routing over satellite networks. *Dissertations & Theses - Gradworks* (2010).

[30] Liu, Z., Zhu, J., Zhang, J. & Liu, Q. Routing algorithm design of satellite network architecture based on sdn and icn. *Int. J. Satellite Commun. Netw.* **38** (2020).

[31] Takkar A (2014). A genetic algorithm for finite state automata. Indian J. Comput. Sci. Eng..

[32] Fischer, D., Basin, D. & Engel, T. Topology dynamics and routing for predictable mobile networks. In *IEEE International Conference on Network Protocols, 2008. ICNP 2008.* (2008).

[33] Bai, J., Lu, X., Lu, Z. & Wei, P. Compact explicit multi-path routing for leo satellite networks. In *Workshop on High Performance Switching & Routing* (2005).

[34] Taleb T, Mashimo D, Jamalipour A, Kato N, Nemoto Y (2009). Explicit load balancing technique for ngeo satellite ip networks with on-board processing capabilities. IEEE/ACM Trans. Netw..

[35] Ömer Korçak, Alagöz, F. & Jamalipour, A. Priority-based adaptive routing in ngeo satellite networks. *Int. J. Commun. Syst.* **20**, 313–333 (2010).

[36] Ruchuan, W. *Satellite Communication Network Routing Technology and Simulation* (Posts & Telecom Press, 2010).

[37] Ekici, E., Akyildiz, I. F. & Bender, M. Datagram routing algorithm for leo satellite networks. In *INFOCOM 2000. Nineteenth Annual Joint Conference of the IEEE Computer and Communications Societies. Proceedings. IEEE* (2000).

[38] Henderson, T. & Katz, R. On distributed, geographic-based packet routing for leo satellite networks. In *IEEE Global Telecommunications Conference* (2000).

[39] Wang, K., Yi, K., Tian, B. & Wu, C. Packet routing algorithm for polar orbit leo satellite constellation network. *Sci. China (Inf. Sci.)* **49**, 103–127 (2006).

[40] De Sanctis, M., Cianca, E. & Ruggieri, M. Ip-based routing algorithms for leo satellite networks in near-polar orbits. In *Aerosp. Conf.* (2003).

[41] Papapetrou, E. & Pavlidou, F.-N. Distributed load-aware routing in leo satellite networks. In *IEEE GLOBECOM 2008–2008 IEEE Global Telecommunications Conference*, 1–5 (2008).

[42] Papapetrou E, Karapantazis S, Pavlidou FN (2007). Distributed on-demand routing for leo satellite systems. Comput. Netw..

[43] Xing, Z., Jun, L., Chundong, D. & Yujing, Z. Security improvement on laor routing protocol. *Journal of Computer Applications* **033**, 1619–1621, 1629 (2013).

[44] Akyildiz IF, Ekici E, Bender M (2002). Mlsr: A novel routing algorithm for multilayered satellite ip networks. IEEE/ACM Trans. Netw..

[45] Lang, X., Zhang, Q., Gui, L., Hao, X. & Chen, H. A novel topology design method for multi-layered optical satellite networks. In Yu, Q. (ed.) *Space Information Networks - 4th International Conference, SINC 2019, Wuzhen, China, September 19-20, 2019, Revised Selected Papers*, vol. 1169 of *Communications in Computer and Information Science*, 87–98 (Springer, 2019). 10.1007/978-981-15-3442-3_8.

[46] Chen, C., Ekici, E. & Akyildiz, I. F. Satellite grouping and routing protocol for leo/meo satellite ip networks. In *the 5th ACM international workshop* (2002).

[47] Lee, J. & Kang, S. Satellite over satellite (sos) network: a novel architecture for satellite network. In *Proceedings IEEE INFOCOM 2000. Conference on Computer Communications. Nineteenth Annual Joint Conference of the IEEE Computer and Communications Societies (Cat. No.00CH37064)* (2002).

[48] Yuan, R. & Wang, R. C. Qos routing based on mobile agent for leo satellite ip networks. *J. China Univ. Posts Telecommun.* 61–67 (2009).

[49] Yanhui, P., Tao, W., Yang, W. & Wenhao, W. Trust-based authentication routing protocol for satellite network. *J. Comput. Appl.* **31** (2011).

[50] Yu, Z., Zhou, H. & Wu, Z. A trust-based secure routing protocol for multi-layered satellite networks. In *2012 IEEE International Conference on Information Science and Technology*, 313–317 (2012).

[51] Zhang, H. *The Research on Dynamic Routing Algorithm and Security Mechanism for Satellite Network Based on Mobile Agent*. Ph.D. thesis, Nanjing University of Posts and Telecommunications (2011).

[52] Rischke, J. & Sossalla, P. Machine learning for routing. *Comput. Commun. Networks* (2021).

[53] Fadlullah, Z. M., Mao, B., Tang, F. & Kato, N. Value iteration architecture based deep learning for intelligent routing exploiting heterogeneous computing platforms. *IEEE Trans. Comput.* (2019).

[54] Zhenyu N (2018). Distributed routing strategy based on machine learning for leo satellite network. Wirel. Commun. Mobile Comput..

[55] Liu, C., Xu, M., Geng, N. & Zhang, X. A survey on machine learning based routing algorithms. *J. Comput. Res. Dev.* 671–687 (2020).

[56] Geyer, F. & Carle, G. Learning and generating distributed routing protocols using graph-based deep learning. In *Proceedings of the 2018 Workshop on Big Data Analytics and Machine Learning for Data Communication Networks*, Big-DAMA ’18, 40–45 (Association for Computing Machinery, New York, NY, USA, 2018). 10.1145/3229607.3229610.

[57] Boyan, J. A. & Littman, M. L. Packet routing in dynamically changing networks: A reinforcement learning approach. *Adv. Neural Inf. Process. Syst.* **6** (1993).

[58] Hu T, Fei Y (2010). Qelar: A machine-learning-based adaptive routing protocol for energy-efficient and lifetime-extended underwater sensor networks. IEEE Trans. Mob. Comput..

[59] Choi, S. & yan Yeung, D. Predictive q-routing: A memory-based reinforcement learning approach to adaptive traffic control. In *In Advances in Neural Information Processing Systems 8 (NIPS8*, 945–951 (MIT Press, 1996).

[60] Kumar, S. & Miikkulainen, R. Dual reinforcement q-routing: An on-line adaptive routing algorithm. In *In Proceedings of the Artificial Neural Networks in Engineering Conference (St. Loius*, 231–238 (ASME Press, 1997).

[61] Basagni, S., Valerio, V. D., Gjanci, P. & Petrioli, C. Finding marlin: Exploiting multi-modal communications for reliable and low-latency underwater networking. In *IEEE INFOCOM 2017 - IEEE Conference on Computer Communications* (2017).

[62] A, S. B., B, V. D. V., B, P. G. & B, C. P. Marlin-q: Multi-modal communications for reliable and low-latency underwater data delivery. *Ad Hoc Networks* **82**, 134–145 (2019).

[63] Kipf TN, Welling M (2017). Semi-supervised classification with graph convolutional networks.

[64] Rusek, K., Suárez-Varela, J., Mestres, A., Barlet-Ros, P. & Cabellos-Aparicio, A. Unveiling the potential of graph neural networks for network modeling and optimization in sdn. In *the 2019 ACM Symposium* (2019).

[65] Kreutz, D., Ramos, F. M. V., Verissimo, P. E., Rothenberg, C. E. & Uhlig, S. Software-defined networking: A comprehensive survey. *Proceedings of the IEEE* **103** (2014).

[66] Bosshart, P. *et al.* Forwarding metamorphosis: Fast programmable match-action processing in hardware for sdn. In *Acm Sigcomm Conference on Sigcomm* (2013).

[67] Bosshart P, Daly D, Izzard M, Mckeown N, Walker D (2013). Programming protocol-independent packet processors. ACM SIGCOMM Comput. Commun. Rev..

[68] Bhuyan MH, Bhattacharyya DK, Kalita JK (2014). Network anomaly detection: Methods, systems and tools. IEEE Commun. Surv. Tutor..

[69] Zhang T (2020). Application of time-varying graph theory over the space information networks. IEEE Network.

[70] Skarding, J., Gabrys, B. & Musial, K. Foundations and modelling of dynamic networks using dynamic graph neural networks: A survey (2020). arXiv:2005.07496.

[71] Ranshous S (2015). Anomaly detection in dynamic networks: A survey. Wiley Interdiplinary Reviews Computational Stats.

[72] Aggarwal, C. C., Zhao, Y. & Yu, P. S. Outlier detection in graph streams. In *Proceedings of the 27th International Conference on Data Engineering, ICDE 2011, April 11-16, 2011, Hannover, Germany* (2011).

[73] Ranshous, S., Harenberg, S., Sharma, K. & Samatova, N. F. A scalable approach for outlier detection in edge streams using sketch-based approximations. In *Proceedings of the 2016 SIAM International Conference on Data Mining* (2016).

[74] Manzoor, E., Milajerdi, S. M. & Akoglu, L. Fast memory-efficient anomaly detection in streaming heterogeneous graphs. In *the 22nd ACM SIGKDD International Conference* (2016).

[75] Yu, W., Cheng, W., Aggarwal, C. C., Zhang, K. & Wang, W. Netwalk: A flexible deep embedding approach for anomaly detection in dynamic networks. In *the 24th ACM SIGKDD International Conference* (2018).

[76] Guo J-Y, Li R-H, Zhang Y, Wang G-R (2020). Graph neural network based anomaly detection in dynamic networks. J. Softw..

[77] Chandola V, Banerjee A, Kumar V (2009). Anomaly detection: A survey. ACM Comput. Surv..

[78] Ahmad, S., Lavin, A., Purdy, S. & Agha, Z. Unsupervised real-time anomaly detection for streaming data. *Neurocomputing* (2017).

[79] Fuertes, S., Picart, G., Tourneret, J. Y., Chaari, L. & Richard, C. Improving spacecraft health monitoring with automatic anomaly detection techniques. In *International Conference on Space Operations* (2015).

[80] Hundman, K., Constantinou, V., Laporte, C., Colwell, I. & Soderstrom, T. Detecting spacecraft anomalies using lstms and nonparametric dynamic thresholding. In *the 24th ACM SIGKDD International*, 387–395 (2018).

[81] Omeara, C., Schlag, L., Faltenbacher, L. & Wickler, M. Athmos: Automated telemetry health monitoring system at gsoc using outlier detection and supervised machine learning. In *14th International Conference on Space Operations* (2016).

[82] Arbon, E. & Smet, P. Anomaly detection in satellite communications networks using support vector machines. In *Aiaa International Communications Satellite Systems Conference & Exhibition* (2013).

[83] O’Shea, T. J., Clancy, T. C. & McGwier, R. W. Recurrent neural radio anomaly detection (2016). arXiv:1611.00301.

[84] Malhotra, P., Vig, L., Shroff, G. & Agarwal, P. Long short term memory networks for anomaly detection in time series. In *23rd European Symposium on Artificial Neural Networks, Computational Intelligence and Machine Learning, ESANN 2015* (2015).

[85] Malhotra, P. *et al.* Lstm-based encoder-decoder for multi-sensor anomaly detection (2016). arXiv:1607.00148.

[86] Gunn, L., Smet, P., Arbon, E. & Mcdonnell, M. D. Anomaly detection in satellite communications systems using lstm networks. In *2018 Military Communications and Information Systems Conference (MilCIS)* (2018).

[87] Shin, Y. *et al.* ITAD: Integrative Tensor-Based Anomaly Detection System for Reducing False Positives of Satellite Systems, 2733–2740 (Association for Computing Machinery, New York, NY. *USA*10.1145/3340531.3412716 (2020).

[88] Eswaran, D., Faloutsos, C., Guha, S. & Mishra, N. Spotlight: Detecting anomalies in streaming graphs. In *the 24th ACM SIGKDD International Conference* (2018).

[89] Wu Q, Fu D, Shen B, Chen Y (2020). Semantic service search in it crowdsourcing platform: A knowledge graph-based approach. Int. J. Softw. Eng. Knowl. Eng..

[90] Wang, Y., Chen, Q., He, C., Liu, H. & Wu, X. Knowledge base question answering system based on knowledge graph representation learning. In *ICIAI 2020: 2020 the 4th International Conference on Innovation in Artificial Intelligence* (2020).

[91] Lee, D., Oh, B., Seo, S. & Lee, K. News recommendation with topic-enriched knowledge graphs. In d’Aquin, M., Dietze, S., Hauff, C., Curry, E. & Cudré-Mauroux, P. (eds.) *CIKM ’20: The 29th ACM International Conference on Information and Knowledge Management, Virtual Event, Ireland, October 19-23, 2020*, 695–704 (ACM, 2020). 10.1145/3340531.3411932.

[92] Lan, L. T. H., Tuan, T. M., Ngan, T. T., Le, H. S. & Hai, P. V. A new complex fuzzy inference system with fuzzy knowledge graph and extensions in decision making. *IEEE Access* **PP**, 1–1 (2020).

